# Vitality tests for pulp diagnosis of adjacent teeth following sinus floor elevation: a prospective study

**DOI:** 10.1007/s00784-025-06440-z

**Published:** 2025-06-25

**Authors:** Jochen Tunkel, Shengchi Fan, Anita Kloss-Brandstätter, Frank R. Kloss, Henning Staedt, Frederic Kauffmann, Diana Heimes, Peer W. Kämmerer

**Affiliations:** 1 Private Practice for Oral Surgery and Periodontology, Königstraße 19, 32545 Bad Oeynhausen, Germany; 2 Department of Oral and Maxillofacial Surgery, University Medical Center Mainz, Augustusplatz 2, 55131 Mainz, Germany; 3 Department of Engineering & IT, Carinthia University of Applied Sciences, Europastraße 4, Villach, 9524 Austria; 4 Private Clinic for Oral- and Maxillofacial Surgery, Kärntnerstraße 62, Lienz, 9900 Austria; 5 Department of Prosthodontics and Materials Science, University of Rostock, Stempelstraße 13, 18057 Rostock, Germany; 6 Department of Periodontology, Faculty of Health, Witten/Herdecke University, Alfred-Herrhausen-Str. 50, 58455 Witten, Germany

**Keywords:** Sinus floor augmentation, Pulp vitality, Dental implant placement, Maxillary sinus, Neurovascular supply, Bone grafting

## Abstract

**Objectives:**

The Schneiderian membrane is elevated to place bone graft material during sinus floor augmentation. Since the teeth’s vascular and neurovascular supply passes through the maxillary sinus, this procedure may lead to sensitivity loss in adjacent teeth. This study is the first to prospectively analyze the vitality of adjacent teeth after sinus floor elevation.

**Materials and methods:**

Data were collected between 2015 and 2023 from 158 patients, who underwent maxillary sinus floor augmentation and implant placement. A total of 378 teeth were examined for vitality using cold testing at the following time points: (1) before sinus floor elevation, (2) 2 weeks, (3) 4 months, (4) 8 months, and (5) 12 months post-surgery. Follow-up duration ranged from 12 to 32 months.

**Results:**

Two teeth in two patients showed no vitality before surgery, but became vital postoperatively. In 3 teeth (0.79%), pulp vitality loss was recorded 2 weeks after surgery, which was restored at the 4-month follow-up. These teeth remained vital after one year. The pre-and postoperative pulp vitality comparison showed no statistically significant difference (*p* = 0.625).

**Conclusions:**

This first prospective observational study demonstrated that a few patients experienced temporary pulp sensitivity loss immediately post-surgery, which resolved within four months.

**Clinical relevance:**

Pulp sensitivity loss due to vascular disruption during sinus floor augmentation is rare and usually temporary. Despite potential vascular disruption, sinus floor augmentation procedures do not appear to cause permanent pulp sensitivity loss.

**Graphical Abstract:**

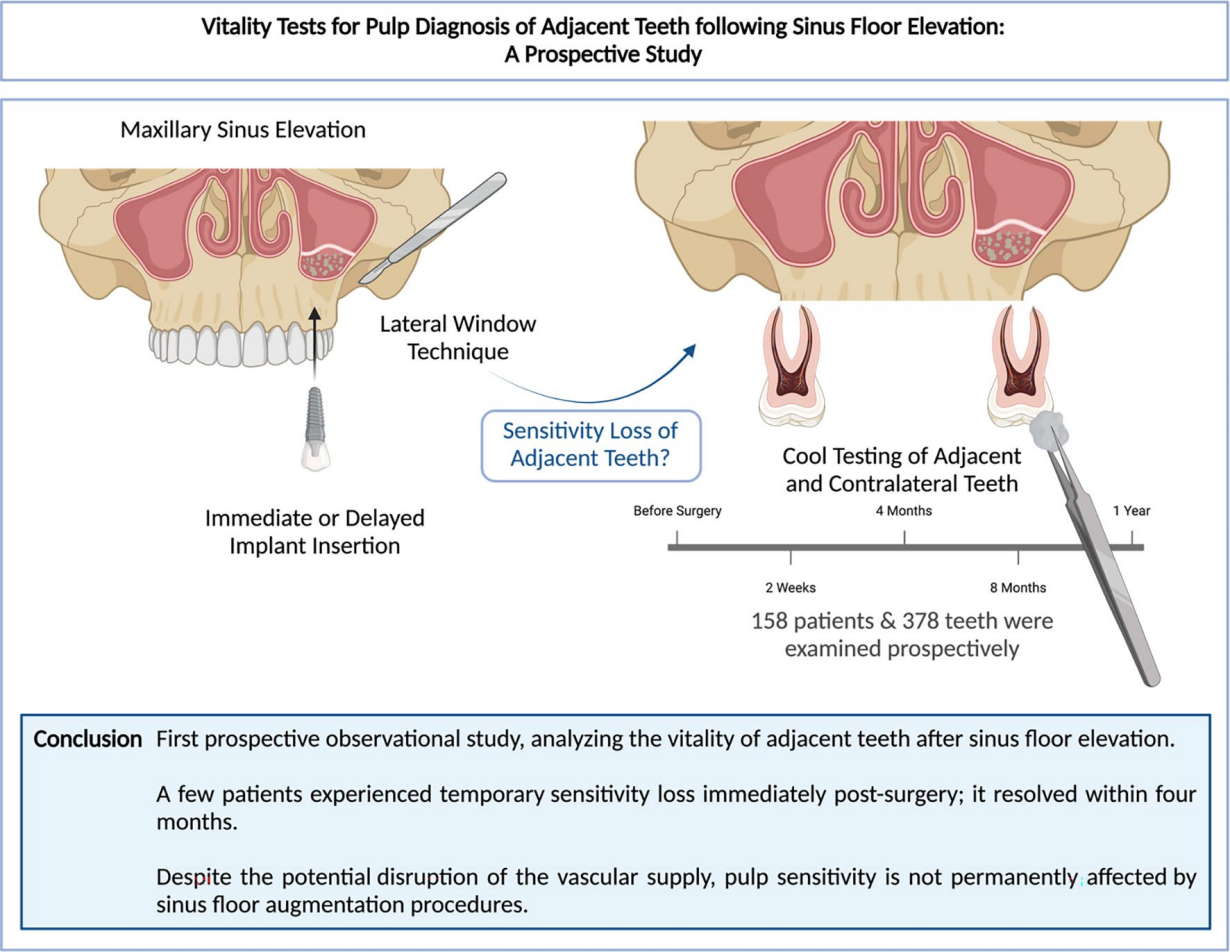

## Introduction

Inadequate bone volume in the posterior maxilla often challenges implant placement and restoration. This deficiency frequently arises from previous infections or natural maxillary sinus pneumatization post-tooth extraction [1]. In 1980, Boyne and James pioneered maxillary sinus floor augmentation via the lateral wall, utilizing grafting material from the iliac crest [2]. Summers later introduced transcrestal osteotome sinus floor elevation in 1994, offering a less invasive alternative, particularly beneficial for alveolar ridges measuring between 5 and 10 mm [3]. Substantial clinical evidence supports sinus augmentation as a gold standard procedure with consistently high success rates [4–6].

Complications, notably Schneiderian membrane perforation during surgical procedures, pose considerable risks for subsequent maxillary sinusitis or loss of grafted material [7]. However, a recent systematic review found that membrane perforation does not significantly impact dental implant survival rates [8].

Despite this, research assessing the effects of membrane elevation and bone augmentation on neighboring teeth remains scarce. Only one case series has explicitly investigated this matter, documenting instances of non-vital pulp observed after sinus lift augmentation [9]. Factors contributing to such occurrences include surgical technique, maxillary sinus anatomy intricacies, and the interplay between root morphology and alveolar height [9]. Beck et al. found that for teeth projecting laterally over the sinus or closely associated with the sinus floor, the incidence of tooth devitalization was less than 0.7% [10]. The latter retrospective study was based on radiographic imaging supplemented by patient medical records. However, detecting devitalized teeth post-sinus augmentation is challenging without regular check-ups, necessitating specific clinical intervention protocols. Factors affecting the effectiveness of sensibility tests in necrotic teeth include patient subjectivity, alterations in pain threshold, and transient paresthesia, which can persist for up to 6 months post-injury, leading to false-negative test results [7]. To address this gap, a prospective observative study with a follow-up of at least one year was designed to evaluate the relevance of loss of pulp vitality following various maxillary sinus augmentation techniques.

## Materials and methods

### Study design and patients

This study was conducted as a prospective observational, non-interventional clinical trial involving patients who sought treatment at the Department of Oral and Maxillofacial Surgery, University Medical Center Mainz, and two private practices specialized in Implantology and Periodontology in Bad Oeynhausen and Esslingen, both Germany, from 2015 to 2023. The Ethics Committee of Rhineland-Palatinate, Germany, approved this research (Approval No. 17687).

Participants underwent comprehensive extra-oral and intra-oral assessments. The extent of bone resorption at sites of tooth loss was evaluated using pre-operative cone-beam computed tomography (CBCT) scans. Each participant underwent sinus floor elevation procedures, and the vitality of teeth adjacent to the elevated sinus was assessed with a follow-up duration of at least one year.

### Inclusion criteria

All participants provided written informed consent. Each patient included in the study was scheduled for sinus floor augmentation, followed by implant placement and the provision of implant-supported fixed restorations. To ensure anatomical relevance, only adjacent teeth within one to two positions mesial and distal to the augmented site were included in the analysis. Teeth were included irrespective of whether their roots radiographically protruded into the sinus cavity or were located at a slight distance from the sinus floor, reflecting the clinical variability of maxillary posterior dentition.

**Fig. 1:**
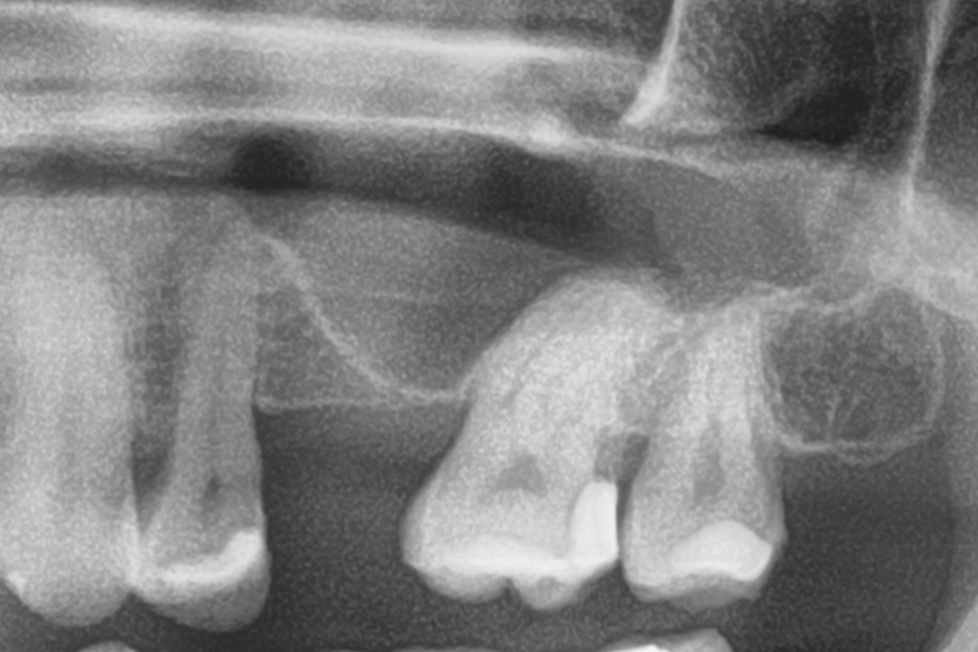
Preoperative radiograph with a planned implant in regio 26

**Fig. 2:**
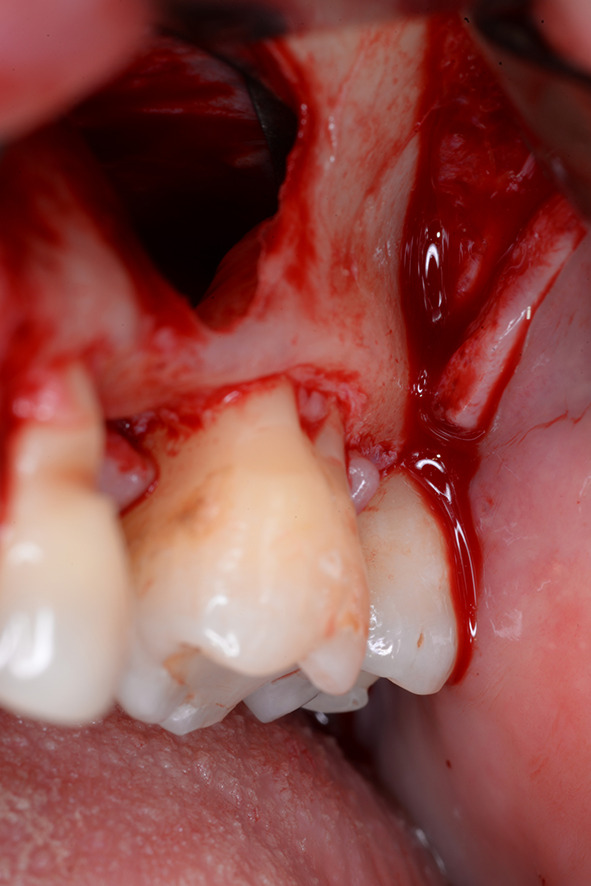
Intraoperative view after a transcrestal approach after elevation of the Schneiderian membrane and before filling the cavity with a bone substitute material. The mesial root of tooth 27 is visible

**Fig. 3:**
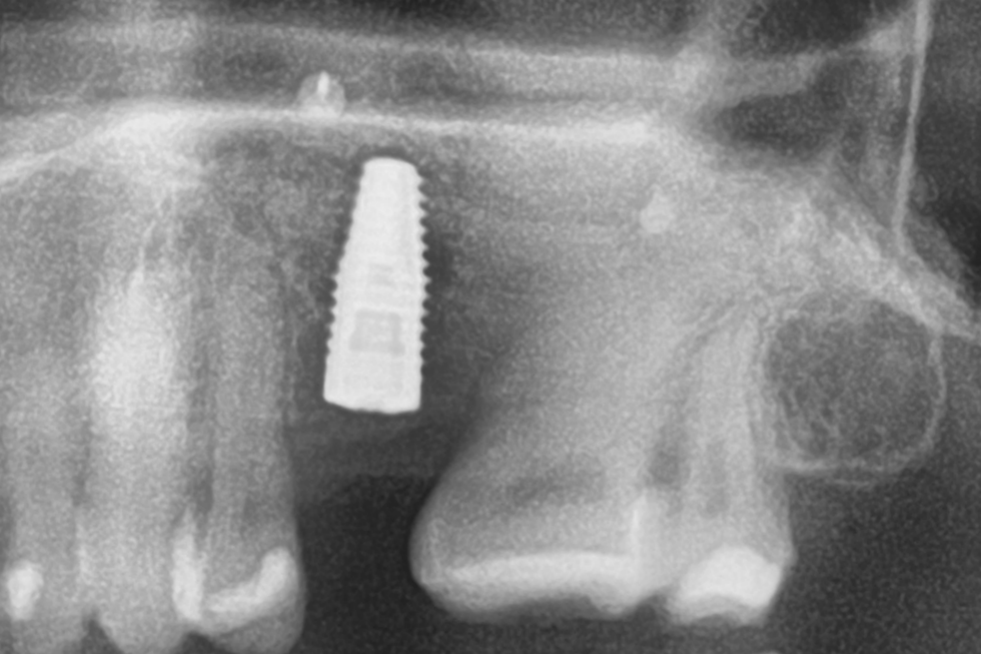
Postoperative radiograph after insertion of the implant

**Fig. 4:**
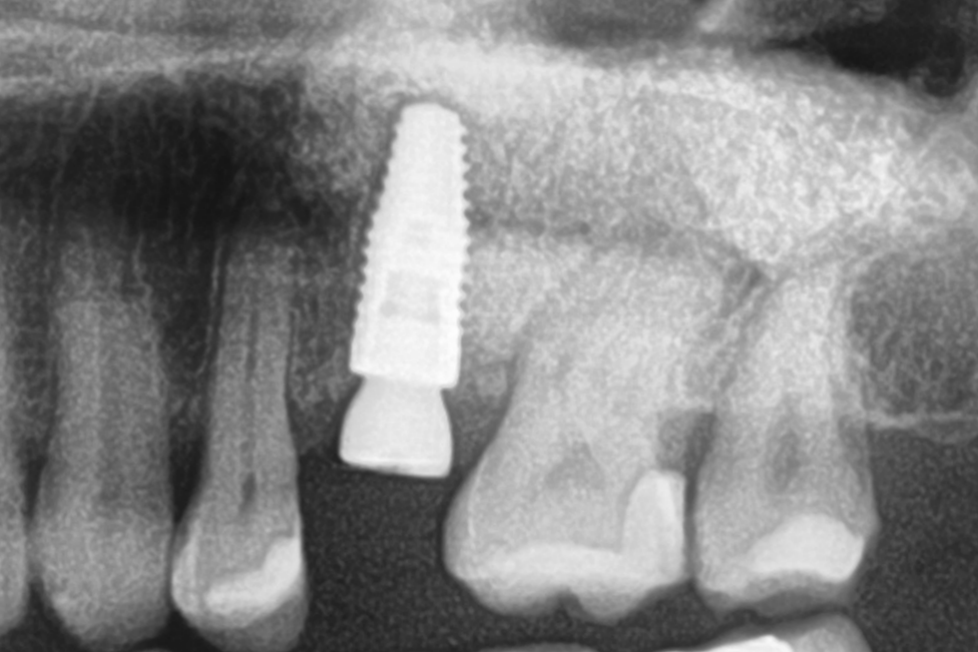
Radiograph after a subgingival healing phase of eight months and insertion of a transgingival abutment

**Fig. 5:**
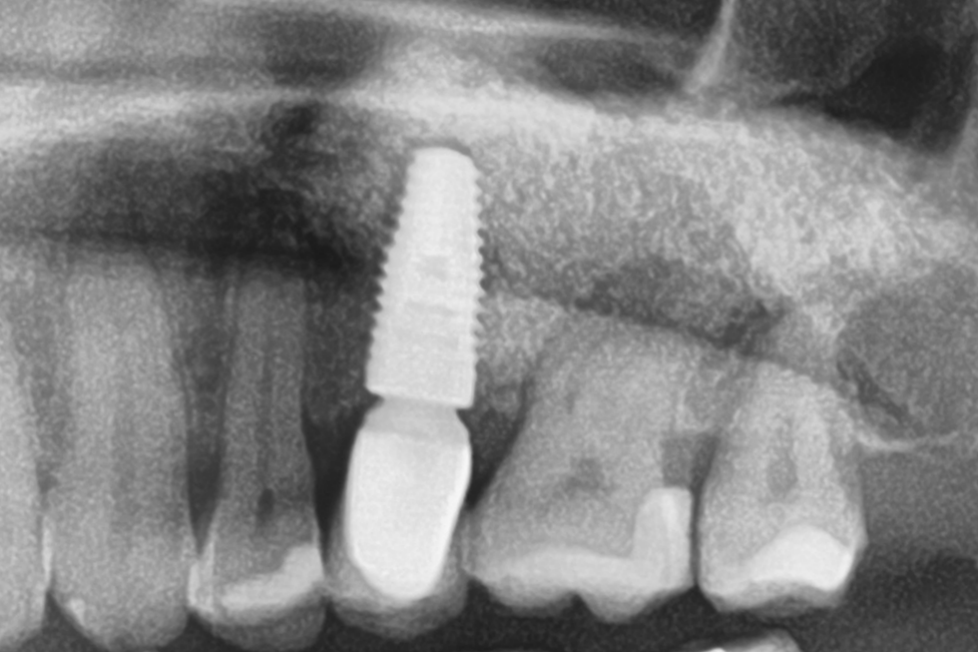
Follow-up radiograph after one year of healing

Participants were categorized based on the amount of residual alveolar bone and the corresponding surgical approach:

1. Patients with a single or multiple tooth edentulous maxilla and 0–4 mm of remaining alveolar bone underwent open sinus lift via lateral maxillary sinus antrostomy followed by delayed implant placement (Figs. 1, 2, 3, 4 and 5).

2. Patients with a single or multiple tooth edentulous maxilla and 5–10 mm of remaining alveolar bone received an open sinus lift via lateral maxillary sinus antrostomy and immediate implant placement.

### Exclusion criteria

The study excluded participants based on the following criteria:

1. Local or systemic contraindications to implant placement (f. e. active periodontitis, acute infections, high-dose therapy with antiresorptive agents, chemotherapy, platelet aggregation inhibitors, active cancer).
2. Untreated maxillary sinusitis, presence of a maxillary sinus cyst, or poor oral hygiene.
3. Heavy smoking (more than 20 cigars per day).

### Surgical procedure

Patients underwent the procedures under local anesthesia. The surgical approach involved making crestal and posterior vestibular releasing incisions to raise full-thickness mucoperiosteal flaps. This was essential for adequate exposure of the posterior alveolar ridge and the lateral wall of the maxillary sinus, particularly during the open sinus lift via lateral maxillary sinus antrostomy. The lateral window technique, through a modified Caldwell-Luc approach, was employed. This technique includes creating osteotomies to fashion a bony window, either removed or medially rotated, ensuring the sinus membrane remains intact. Membrane elevation was performed using broad-based instruments or curettes, elevating the membrane slightly above the level of the superior osteotomy line. The resultant cavity was filled with bone graft material, which was loosely packed to avoid over-compression. The graft materials varied, including xenograft, allogeneic bone, or a combination thereof. Additional particulate bone was harvested from multiple intraoral sites such as the maxillary tuberosity, lateral wall of the maxilla, mandibular symphysis, ramus, posterior maxilla, and the site of the mandibular third molar. If pre-operative bone height was over 5 mm, implants were placed immediately following sinus cavity filling. If conditions did not permit immediate implant placement, the site was primarily closed, and staged implant placement was scheduled 4 to 6 months post-sinus lift. Three experienced surgeons conducted all operative procedures. Patients were prescribed a one-day course of antibiotics (amoxicillin or clindamycin), analgesics (ibuprofen and/or acetaminophen), and a chlorhexidine 0.2% mouthwash solution (three times daily for two weeks) to enhance recovery and minimize infection risks.

### Second stage procedure and follow-up evaluation

The second-stage surgery, either for exposing the implants or for implant placement, was performed 3 to 6 months after the initial treatment. In the case of implant placement, a crestal incision was conducted to raise a circumscribed full-thickness mucoperiosteal flap. Implant bed preparation was performed according to the company’s drilling protocol and implants were inserted. The site was primarily closed for later implant exposure. Implant exposure involved uncovering the implants to prepare them for the subsequent prosthetic phase. Based on the clinical scenario and available technology, final prosthetic restorations were created by taking impressions using either a coping transfer technique or an intraoral scanner. Patients received either fixed crowns or bridges as their final restoration. Follow-up evaluations were systematically scheduled to monitor the implants’ success and the surrounding tissues’ health.

### Endpoint of the study

Before conducting the sinus floor elevation procedures and at each follow-up time point (2 weeks, 4 months, 8 months, and 12 months after the surgery), the vitality of teeth adjacent to the augmented sites was assessed using cold testing. Teeth located within one to two positions mesial and distal to the augmented site were included in this assessment to reflect anatomical proximity. To minimize the likelihood of false results, each tooth underwent testing twice during every observation period. For cold testing, cold spray (PCKW-free) with an exit temperature of -40 °C (propane/butane mixture, Henry Schein Dental, item number 9001384, Melville, New York, United States) was applied on a foam pellet that was immediately placed on the tooth without any contact to the adjacent mucosa. The pellet was removed after the patient reported of a cold feeling on the tooth or at the latest 15 s.

### Statistical analysis

According to the current literature, tooth vitality loss after maxillary sinus augmentation is a rare event. There is only one retrospective analysis that explicitly measured the frequency of pulp vitality loss in patients after this surgical intervention. Beck et al. reported a frequency of 0.7% in 357 teeth [10]. Furthermore, there is a case series reporting of a loss of pulp vitality after maxillary sinus augmentation in three patients [9]. To conduct a sample size calculation, data from studies analyzing pulp vitality loss after Le Fort I osteotomy, which is a distinctly invasive procedure, were used. In 1989 Vedtofte et al. reported a pulp vitality loss in 8% of patients after one year and in 6% of cases after five years (617 examined teeth) [11]. A similar frequency has been reported by Saeed and colleagues. The frequency of negative cold testing was 5% six months after surgery (26 patients) [12]. Sat et al. measured an incidence of 3.7% after 12 months [13]. Regarding the heterogeneous data, a mean assumed incidence of 3% was assumed because of the, on the one hand, retrospective study design by Beck et al., which might underestimate the frequency of pulp vitality loss and, on the other hand, the potential higher frequency of events in more invasive surgeries like the Le Fort I osteotomy. Since no data is reporting the frequency of physiological pulp vitality loss in healthy teeth during one year of observation, we supposed an incidence of 0.1%. Assuming an incidence of pulp vitality loss after maxillary sinus augmentation of 3% and a physiological vitality loss during the observation period of 0.1%, a minimum of 205 teeth had to be examined (power 80% and level of significance 5%). Sample size planning was performed with RStudio using the package “pwr”.

Data were managed using Excel (Microsoft, Redmond, WA), and statistical analysis was conducted using the SAS statistical package (SAS 9.3, SAS Institute Inc., Cary, NC, USA). Descriptive statistical analysis was performed for continuous variables (mean, standard deviation, median, minimum, and maximum) and categorical variables (absolute frequency and percentage). McNemar’s Test was employed to assess the association of proportions between preoperative and postoperative pulp vitality, and a p-value of ≤ 0.05 was considered statistically significant.

## Results

### Patient characteristics

Over 8 years (2015 to 2023), data were collected from 180 patients who underwent maxillary sinus floor augmentation and implant placement. However, 22 patients did not complete the pre-operative pulp vitality examination. Therefore, 158 patients (62 males, 96 females; mean age 59.3 years; age range: 35–95 years) with 378 teeth were examined after sinus augmentation with a follow-up duration of at least one year (12–32 months). Among the 158 patients, 21 (13.3%) had systemic diseases, and 99 (62.7%) had a history of periodontitis. Detailed characteristics information is provided in Table 1.

**Table 1:** Medical characteristics of 158 patients

Patient characteristics	Periodontitis	
	-	+
Patients (n; %).	59 (37.3%)	99 (62.7%)
Males (n; %)	62 (39.2%)	96 (60.8%)
Age (Mean ± SD)	53.7 ± 8.25	63.4 ± 7.7
Augmentation site (R/L)	51/30	78/45
Followed up teeth	168	210
With systemic disease (n; %)	6 (3.8%)	15 (9.5%)
Smokers (n; %)	17 (10.8%)	21 (13.3%)

### Augmentation sites and healing time

This study utilized different surgical approaches, employing various bone augmentation material for maxillary sinus augmentation. Thirty patients underwent bilateral sinus augmentation, while 128 underwent unilateral sinus augmentation, with 102 on the right side and 26 on the left, totaling 188 sinus floor elevation procedures. Implants were placed simultaneously with augmentation in 101 out of 188 cases. The average healing time for augmentation was 5.67 ± 1.65 months, and for implants placed in the second stage, it was 5.64 ± 2.20 months. Among the procedures, 22 out of 188 cases (11.7%) exhibited visible sinus membrane perforation during elevation; however, all perforations were less than 2 mm and were covered by a resorbable collagen membrane. A total of 210 implants were inserted following sinus floor elevation procedures, with a cumulative implant survival rate of 94.3%, including 12 losses out of 210 implants (5.7%), comprising two early losses (0.95%) and ten losses due to peri-implantitis out of 208 implants (4.8%).

### Pulp vitality testing

A total of 378 teeth pulp vitality was examined from 158 patients. Two teeth in two patients were examined with no pulp vitality before the operation, and positive results were obtained two weeks after sinus elevation procedures. Another three teeth (0.79%) displayed no pulp vitality postoperatively but turned positive at the 4-month follow-up. Those teeth remained vital after one year of follow-up, suggesting that the initial examinations yielded false-negative results due to human error. The association of proportions between preoperative and postoperative pulp vitality was analyzed using McNemar’s Test, yielding a p-value of 0.625. This suggests that the difference between the paired proportions before and after sinus floor augmentation is not statistically significant.

## Discussion

The study aimed to investigate the pulp vitality of adjacent teeth following sinus floor elevation, using cold testing over a follow-up period of 12 months post-surgery. The results indicated that while two teeth displayed a loss of vitality immediately following the surgery, all showed recovery within four months, with no long-term negative effects observed. There was no statistically significant difference between preoperative and postoperative pulp vitality, suggesting that the risk of permanent vitality loss is minimal.

Known intraoperative and postoperative complications of maxillary sinus elevation procedures are reported to be the perforation of the Schneiderian membrane, bleeding, graft displacement, hemoptysis, sinusitis, sinus congestion, and infection [14]. The devitalization of neighboring teeth might be another unwanted complication that is substantiated by scarce evidence only [9, 10]. The suspected main reasons for this event are the disrupted blood flow of the alveolar antral artery to adjacent teeth, even if a collateral circulation within the maxillary alveolar bone was reported, or damage to the sensory nerves [10]. The maxillary sinus receives blood supply from multiple arteries, including the infraorbital artery, greater palatine artery, lesser palatine artery, sphenopalatine artery, and the posterior superior alveolar artery [15]. The design of the lateral approach for sinus floor augmentation needs to be adapted to the course of these vessels as their anastomosis forms a vascular arcade supplying the Schneiderian membrane and the anterolateral wall of the maxillary sinus. This anastomosis is reported to be either intraosseous, submucosal, or subperiosteally. Its course can be visualized by computed tomography or cone-beam computed tomography as a groove of the infero-lateral wall of the maxillary sinus [16]. A previous anatomical study was able to show, that the dentate maxilla is very densely vascularized while the blood supply is reduced with patient age and bone atrophy [17]. It is suggested, that the reduction of the cancellous proportion of the alveolar ridge, which typically is supplied by centro-medullary and periosteal vessels in the dentate maxilla, leads to a diminution to only a periosteal blood supply in the atrophic bone, which might aggravate the negative effects of any vascular damage. Although sinus osteotomy and membrane elevation during sinus elevation procedures have not been shown to disrupt the blood supply to the dental plexus [18], potential damage to these arteries remains a concern.

The maxillary teeth are innervated by the second branch of the trigeminal nerve branching into the anterior, middle, and posterior superior nerve [19]. They form a dense neural network innervating the incisor, canine, premolar, and first molar region as well as the nasal cavity [16]. Even though sensitivity loss after disruption of the teeth’s neural connection and blood supply could be assumed, there are case reports in the literature describing unintentional partial amputation of multirooted teeth during sinus operation or exposure of an impacted canine, where the teeth remained vital for up to 3 to 4 years [20]. Also, maxillary teeth devitalization has been reported to be a rare event even in more invasive procedures such as Caldwell-Luc surgery [21–23]. Even in more extensive surgeries like the Le Fort I osteotomy in patients with facial deformities, loss of pulp vitality is a rather rare event. In 1989 Vedtofte et al. analyzed 617 teeth and reported a pulp vitality loss in 8% of patients after one year and in 6% of cases after five years [11]. A similar frequency has been reported by Saeed and colleagues. The frequency of negative cold testing was 5% six months after surgery in 26 patients [12] and Sato et al. measured an incidence of 3.7% after 12 months [13]. To date, there is only one study that retrospectively examined pulp vitality after maxillary sinus augmentation in 357 teeth based on radiographic imaging supplemented by patient medical records. Here the incidence of tooth devitalization was less than 0.7% [10]. Furthermore, Romanos et al. reported three cases in which pulp vitality was lost after sinus augmentation surgery [9]. Cold spray testing was utilized in this study to assess the pulp vitality of teeth adjacent to the maxillary sinus before and after sinus floor elevation. While this is a widely used and generally reliable method for evaluating pulp vitality [24, 25], there are several limitations that should be considered when interpreting the results of this study. One limitation of cold testing is its reliance on subjective patient responses, which can introduce variability. Another limitation is that cold testing may not accurately reflect the true physiological state of the pulp as a positive response does not guarantee a completely healthy pulp. Additionally, the use of cold spray testing provides only a binary measure (positive or negative response) without offering information on the degree of pulp health or the presence of partial necrosis. Complementary diagnostic tools such as electric pulp testing [26] or laser Doppler flowmetry might enhance the accuracy and reliability of pulp vitality assessments in future studies. Also, this study lacks of precise classification of root–sinus floor proximity. It is plausible that teeth with apices located farther from the sinus floor are less susceptible to neurovascular disturbances during sinus augmentation. While our cohort reflects real-world variability, future studies might include radiographic distance measurements to stratify risk more precisely based on anatomical proximity. However, due to the large number of teeth analyzed (*n* = 378), anatomical variations may have been evenly distributed within the sample, thus minimizing the impact of this limitation on the overall findings.

## Conclusion

While sinus floor elevation may lead to transient pulp vitality loss in a small percentage of cases, this effect is typically reversible within a few months. The procedure does not pose a significant long-term risk to the vitality of adjacent teeth, making it a reliable approach for bone augmentation and implant placement.

## Data Availability

Data are available upon request from the corresponding author.
